# Laparoscopic adrenalectomy: A single center experience

**DOI:** 10.4103/0972-9941.72595

**Published:** 2010

**Authors:** Suresh Kumar, Moley K Bera, Mukesh K Vijay, Arindam Dutt, Punit Tiwari, Anup K Kundu

**Affiliations:** Department of Urology, Institute of Post-Graduate Medical Education and Research (IPGMER) and Seth Sukhlal Karnani Memorial Hospital (SSKM), Kolkata - 700 020, India

**Keywords:** Laparoscopic adrenalectomy, benign adrenal disorders, lesser morbidity

## Abstract

**AIMS:**

To evaluate the efficacy and safety of laparoscopic adrenalectomy in benign adrenal disorders.

**METHODS AND MATERIAL:**

Since July 2007, twenty patients have undergone laparoscopic adrenalectomy for various benign adrenal disorders at our institution. Every patient underwent contrast enhanced CT-abdomen. Serum corticosteroid levels were conducted in all, and urinary metanephrines, normetanephrines and VMA levels were performed in suspected pheochromocytoma. All the patients underwent laparoscopic adrenalectomy via the transperitoneal approach.

**RESULTS:**

The patients were in the age range of 18-57 years, eleven males and nine females, seven right, eleven left, two bilateral. The mean operative time was 150 minutes (120-180), mean hospital stay four days (3-5), mean intraoperative blood loss 150 ml and mean post-operative analgesic need was for 36 (24-72) hours. One out of twenty-two laparoscopic operations had to be converted into open adrenalectomy due to intra-operative complications.

**CONCLUSIONS:**

Laparoscopic adrenalectomy is a safe, effective and useful procedure without any major post-operative complication and is the gold standard for all benign adrenal disorders.

## INTRODUCTION

Traditionally, adrenalectomy is done by an open procedure since 1914, when Sargent performed first planned adrenalectomy.[1] In the modern era of minimally invasive surgery (MIS), laparoscopy is being increasingly used. Laparoscopic adrenalectomy can be performed via the transperitoneal or retroperitoneal route-the former via the lateral or anterior approach and the latter via the posterior approach. Robot-assisted laparoscopic adrenalectomy may also be performed if facilities are available. Each technique has its own advantages and disadvantages. It is now an established fact that different advantages of the laparoscopic approach include less morbidity, better cosmesis, reduction in wound infection, minimal need for analgesia, decreased hospital stay and early return to work.[2–4] We report our experience of laparoscopic adrenalectomy using the transperitoneal approach in 20 patients with different adrenal pathology. This prospective study was conducted in our Urology Department to evaluate the efficacy and safety of laparoscopic adrenalectomy in benign adrenal disorders

## MATERIALS AND METHODS

Between July 2007 and September 2009, a total of 20 patients underwent laparoscopic adrenalectomy at our institution. Exclusion criteria were tumour size > 7 cm and / or suspicion of malignancy on contrast-enhanced CT scan. Peri-operative data were collected and analysed. The total number of patients was 20, Male-eleven, female-nine. Patients were in age ranging from 18-57 years (mean age-38.55 years). Twenty-two laparoscopic adrenalectomies were performed in20 patients (bilateral adrenalectomy in two cases)

In our study, indications for laparoscopic adrenalectomy were pheochromocytoma (n = 8), Conn’s syndrome (n = 1), cortisol secreting adenoma (n = 4), myelolipoma (n = 3) and Ectopic ACTH-dependent adrenal hyperplasia (n = 2). Two cases were incidenteloma (n = 2) detected during investigation for nonspecific backache.

All patients were assessed by history and physical examinations. Contrast-enhanced computed tomogram (CECT)-abdomen was performed in each case. Serum cortisol 8.0 am, 11.0 pm, and 1 mg post-dexamethasone serum cortisol levels were conducted in all patients. Twenty-four hour urinary metanephrines, nor-metanephrines and Vanillylmandelic acid (VMA) levels were performed in suspected pheochromocytoma. Complete hemogram, coagulation profile, and biochemical parameters, including serum electrolytes were completed in all patients. Pre-operative patients profile including various indications of laparoscopic adrenalectomy is summarized in Table 1.

**Table 1:** Pre-operative patients profile

Age and Sex	Side RT / LT	Functional status	Tumour size (cm) on CECT-scan	Indication
22 F	RT	Cortisol (11 pm): raised and post-dexamethasone cortisol raised	3.4	Cortisol secreting adenoma
28 M	BL	Serum Cortisol and ACTH raised, pituitary MRI-normal, Chest CT-normal, high-dose dexamethasone suppression test showed no suppression.	3.2, 3.0	Cushing’s syndrome
18 M	RT	24-hour urinary metanephrines and normetanephrines raised	6.5	pheochromocytoma
55 M	LT	Non-functional	6.0	myelolipoma
52 F	LT	Non-functional	7.0	myelolipoma
36 F	LT	Non-functional	4.2	backache (incidentaloma)
35 F	RT	24-hour urinary metanephrine and normetanephrine raised	5.5 cm	pheochromocytoma
57 M	BL	Ectopic ACTH secretion, secondary to cancer of lung	3.0, 2.8	Cushing’s syndrome
22 M	RT	Cortisol (11 pm): raised and post-dexamethasone cortisol raised	3.8	Cortisol secreting adenoma
35 M	RT	24-hour urinary metanephrines and normetanephrines raised	5.8	pheochromocytoma
32 F	LT	24-hour urinary metanephrines and normetanephrines raised	6.1	Pheochromocytoma
25 M	LT	Cortisol (11 pm): raised and post-dexamethasone cortisol raised	3.3	cortisol secreting adenoma
57 F	LT	Non-functional	6.9	myelolipoma
42 F	LT	24-hour urinary VMA raised	7.0	Pheochromocytoma with neurofibromatosis
33 M	RT	Cortisol (11 pm): raised and post-dexamethasone cortisol raised	3.2	cortisol secreting adenoma
36 F	LT	24-hour urinary VMA, metanephrines and normetanephrines raised	6.5	Pheochromocytoma
43 F	LT	Non-functional	3.0	Backache (incidenteloma)
47 F	RT	24-hour urinary metanephrines and normetanephrines raised	6.8	Pheochromocytoma
40 M	LT	Persistent hypokalemia with aldosterone / rennin ratio-44	2.4	Conn’s adenoma
56 M	LT	24-hour urinary metanephrines and normetanephrines raised	5.6	Pheochromocytoma

RT: right, LT: left, BL: bilateral, M: male, F: female

Patients with pheochromocytoma were treated pre-operatively with prazosin and other antihypertensive and all patients received propranolol one week prior to surgery.

Patients with cortisol secreting adenoma were treated with intravenous hydrocortisone during the day of the operation and on the first post-operative day and further substitution was performed orally, with hydrocortisone tablets.

The Conn’s syndrome patient underwent plasma renin activity and plasma aldosterone level, after correction of hypokalemia. Plasma aldosterone renin ratio was then measured. After measuring the plasma rennin-aldosterone ratio, the patient underwent a sodium loading test with 2 l intravenous normal saline, after which CECT-abdomen was performed.

In case of adrenal hyperplasia with ectopic adrenocorticotropic hormone (ACTH) secretion, one patient underwent pituitary magnetic resonance imaging (MRI), Chest computed tomography (CT) scan and a high-dose dexamethaone suppression test (2 mg every 6 hours for 2 days). The pituitary MRI and Chest CT scan were normal and there was no suppression in serum cortisol. The other patient was a known case of non-small-cell bronchogenic carcinoma with ectopic ACTH secretion. Prior to induction, intravenous third generation cephalosporin was given to all patients. After general anaesthesia, a nasogastric tube and Foley catheter were introduced. Outcome measures included were-operative time, intraoperative open conversion rate, estimated blood loss, post-operative complications, duration and dose of analgesic need and post-operative hospital stay. Patients were placed in the lateral decubitus position with side of the lesion being elevated to 45 degrees.

Right adrenalectomy was usually performed with four ports. After making a skin incision, a primary Camera port 10 mm was placed about 3 cm lateral and cephalad to the umbilicus, using the close method. Two working ports, 5 mm and 10 mm were placed in the midclavicular position, the upper one (5 mm) below the costal margin, and the lower one (10 mm), 10-12 cm below the upper one. Another 5 mm port was placed in the sub-xiphisternal position for liver retraction. A fifth 5 mm port, if required, was placed in the right anterior line, to facilitate retraction. The right colon was mobilised along the line of Toldt. Via sub-xiphisternal port, the liver was retracted using a fan retractor and the triangular ligament was transected. The posterior peritoneum was incised along the surface of the liver, extending from the line of Toldt laterally, up to the inferior vena cava (IVC) medially. Subsequently, the duodenum was mobilised medially, to expose the renal hilum. Hepatodiaphragmatic attachments were identified and dissected. The right adrenal vein entering into the IVC was identified and isolated, and a hem-o-lok clip [Figure 1a] or Ligaclip (depending upon the need) was applied and transected. Subsequently, small superior and inferior adrenal vessels were coagulated and cut with LigaSure, completing the dissection of adrena gland tumour [Figure 1b]

**Figure 1a:**
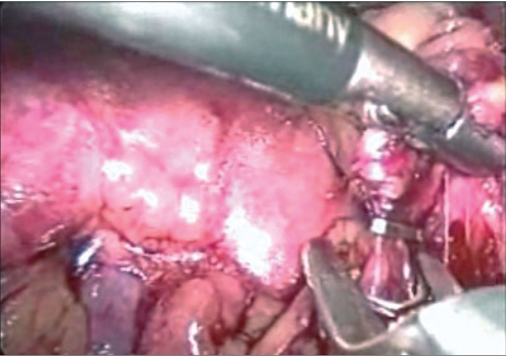
Ligation of right adrenal vein.

**Figure 1b:**
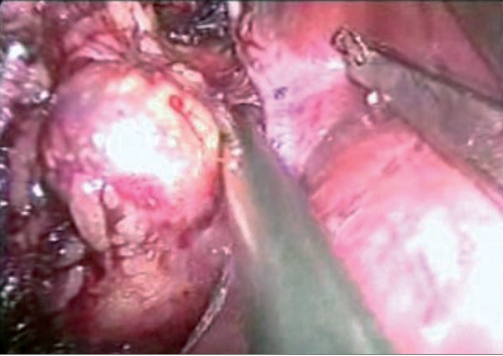
Dissecting adrenal gland tumour.

Usually three ports were used in case of left adrenalectomy. A fourth 5 mm port, if required, was placed in the left mid-axillary line to facilitate the retraction. The colon was mobilised along the line of Toldt, with a release of the splenocolic and phrenocolic ligaments. Subsequently, the lienorenal ligament was incised. After incising the gerota fascia, the upper pole of the kidney was visualised. Dissection along the medial aspect of the kidney was continued till the left renal vein was identified. Next, dissection along the superior aspect of the renal vein identified the left adrenal vein, which was clipped and divided. Sometimes, the gonadal vein helped in identifying the adrenal vein. After controlling the adrenal vein, the superior aspect of the adrenal gland was mobilised and the phrenic vessels supplying the gland were divided. The medial aspect of the adrenal gland was mobilised from the aorta and the vessels supplying the gland were divided using LigaSure. Finally, the lateral attachments of the gland were divided, to free the gland fully from the surrounding tissue.

An abdominal tube drain was inserted in all patients and it was usually removed after 48 hours. For specimen extraction, we either used gloves or an indigenous plastic envelope. The specimen was extracted via a 10 mm port, either by enlarging the incision or via ovum forceps, in pieces. The port sites were closed using the standard technique.

## RESULTS

A total of 22 laparoscopic adrenalectomies were performed, but one required conversion to open, due to uncontrolled intra-operative bleeding from the right adrenal vein (patient 7 in the list). Post-operative complications occurred in three patients: one patient developed port site infection, one patient had atelactasis and one patient post-operative hypotension. All were managed conservatively. Twenty-two laparoscopic adrenalectomies were performed, seven on the right side, eleven on the left side and bilateral in two patients. Mean intra-operative and post-operative blood loss was 150 ml (120-180 ml). Mean operative time was 150 minutes (120-180 minutes). Mean post-operative analgesic need was for 36 hours (24-72 hours), with 50 mg tramadol twice a day. Oral nutrition was resumed after an average of two (1-3) days. Mean hospital stay was four days (3-5), excluding the patient who needed conversion to open adrenalectomy.

Final diagnosis (after histopathology), number of patients in each group and mean tumour size in each group is illustrated in Table 2.

**Table 2:** Adrenal tumour characteristics

Final diagnosis	No. of patients	Mean tumour size (cm)
Cortisol secreting adenoma	4	3.4
Aldosterone secreting adenoma	1	2.4
Adrenocortical hyperplasia	2	3.0
pheochromocytoma	8	6.2
Myelolipoma	3	6.6
Non functional cortical adenoma (Incidentaloma)	2	3.6

## DISCUSSION

Gagner performed the first laparoscopic adrenalectomy in 1991.[5] Pre-operatively, the surgeon was primarily interested in the size of the gland, its vascularity, proximity or involvement of the surrounding structures and functional status.

Contraindications for laparoscopic adrenalectomy, reported in recent literature, are invasive adrenocortical carcinoma, large tumour > 10-12 cm in diameter and malignant ACTH secreting pheochromocytoma with lymphadenopathy and adrenocortical carcinoma with caval thrombus.[6] On account of the aggressive nature of the adrenocortical carcinoma, an open approach allows en block resection and potential removal of the surrounding organs.[7,8] Other absolute contraindications include uncorrectable coagulopathy, severe cardiopulmonary disease and uncontrolled pheochromocytoma. A previous abdominal scar is no longer a contraindication to laparoscopic adrenalectomy.

A relative contraindication to laparoscopic surgery is the tumour size. By initial experience, many laparoscopists used cut off of 5 or 6 cm. Mac Gilliv Ray and Henry recommended an upper size limit of 12 cm for laparoscopic adrenalectomy. Although adrenal masses of up to 10-15 cm are resectable laparoscopically, dissection may be difficult, time consuming and the exposure may not be optimal due to the limited working space. Furthermore, a tumour with a diameter > 6-8 cm carries a risk of malignancy.[9] Hence, we carried out laparoscopic adrenalectomy in tumours measuring up to 7 cm.

**Figure 2:**
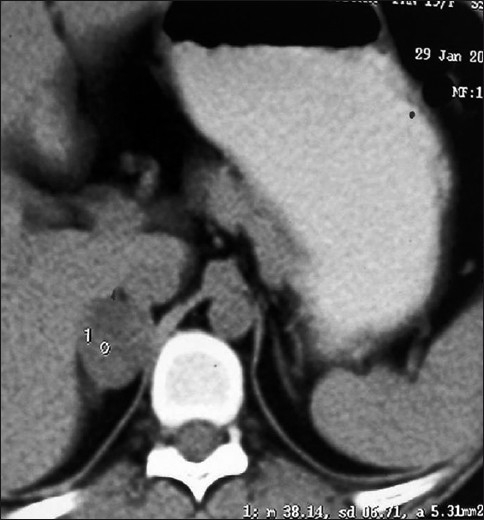
CECT-Right adrenal cortical adenoma.

Radiological imaging contrast-enhanced CT scan or MRI localizes the adrenal mass. On CECT, cortisol secreting adenomas are usually well-defined and small in size, 2-5 cm, homogenous, and show low enhancement with contrast (0-30 HU).[10] Calcification, haemorrhage and necrosis is uncommon in them [Figure 2]. In our study, the mean size was 3.4 cm. Patients demonstrating an excess of cortisol have an increased amount of adipose tissue and some have argued that laparoscopic approach in these patients is most appropriate.[11]

**Figure 3:**
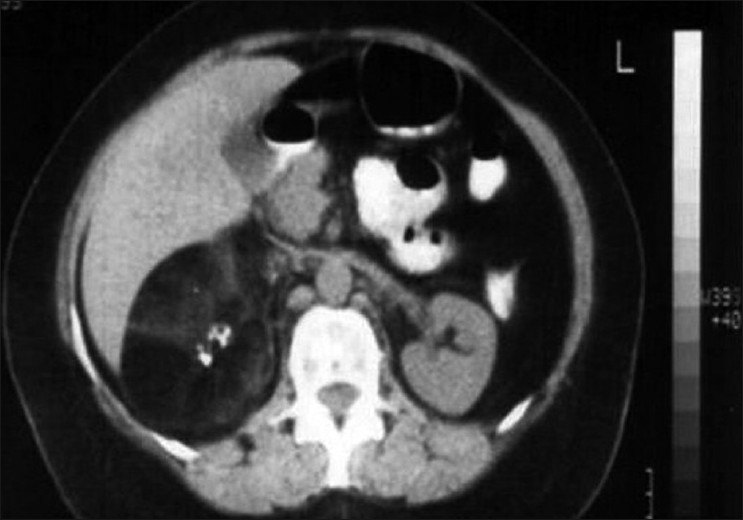
CECT-Right adrenal mass with negative soft tissue density with calcification (myelolipoma).

Adenomyelolipoma are usually well-circumscribed with (-100 to -200 HFU) soft tissue density lesion and may show calcification, which may be diffuse or spotty. In our study, only one patient showed calcification [Figure 3].

Conn’s syndrome can be due to adenomas, adrenal hyperplasia or very rarely due to carcinoma. Adenoma in Conn’s syndrome is typically small, mean diameter 1.5 cm, ranging from 0.5-3.5 cm. Hence, a thin CT scan section (3-5 mm) is mandatory for the reliable detection of adenoma. Accuracy of the CT scan in detecting the aldosteronoma depends upon its size, although with newer scanning techniques (breath-hold thin sections), the overall detection rate is in the range of 80%.[12] In our study, the adenoma was 2.4 cm in size, on a CECT scan.

Adrenal hyperplasia on a CT scan usually shows diffuse thickening and elongation of the adrenal rami or may rarely show prominent glands bilaterally, within normal range.[13] In our study, both patients of ectopic ACTH secretion have shown thickening and elongation of the adrenal rami.

**Figure 4:**
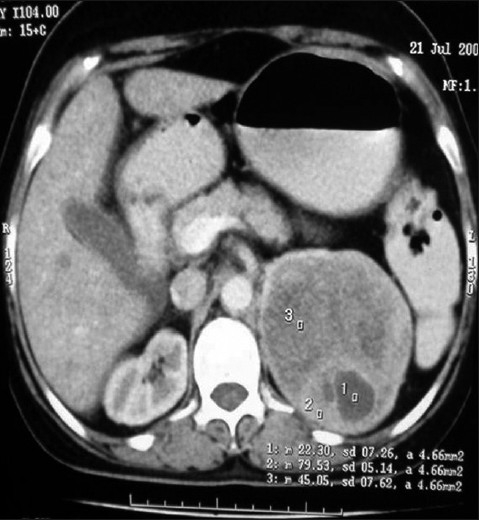
CECT-Left adrenal mass with areas of haemorrhage and necrosis (pheochromocytoma).

Pheochromocytoma are the tumours derived from chromaffin cells that produce and often secrete catecholamines. Highest incidence occurs during the fourth and fifth decade of life and is nearly identical in both the sexes.[14] Pheochromocytoma are usually large and have areas of haemorrhage and necrosis [Figure 4] and may even have fluid-filled levels within them.[13] In multiple endocrine neoplasia (MEN) syndrome patients, tumours are bilateral and tend to be smaller.[15] Generally, intravenous contrast material is not essential for detection of adrenal pheochromocytoma, but good oral contrast for the opacification of the gastrointestinal tract is essential for detecting the retroperitoneal tumour, because unopacified bowel can simulate a para-aortic mass.

In our study, the mean diameter of the tumour, in the case of the pheochromocytoma, was 6.2 cm. Previously pheochromocytoma was considered to be a relative contraindication to laparoscopic surgery, but now laparoscopic adrenalectomy for pheochromocytoma has been performed successfully and reported in several series.[16,17] Regarding pheochromocytoma surgery, it is important to have early stage control of the main adrenal vein, with the intention of reducing blood pressure decompensation, yet it is questionable these days.[18] Approximately 10-15% of the pheochromocytoma are malignant. In order to diagnose malignant behaviour, one must document invasion of the adjacent organs or metastatic disease. The most frequent sites of metastases are the liver, lung, bone, particularly the spine, skull and ribs. There are no absolute, clinical, imaging or laboratory criteria to predict the malignancy. However, patients with malignant disease tend to have a larger tumour and higher urinary metanephrines.[19]

In our series, we came across two patients of incidentaloma during the work-up, for nonspecific backache. One patient had a left adrenal mass of 4.2 cm and another one had a left adrenal mass of 3.0 cm. The final histopathology revealed cortical adenoma in both.

Malignant masses on CT scan are usually large, with irregular margins, inhomogenous density and thick irregular enhancing rims. Central necrosis, calcification and invasion of adjacent structures may be present in them. Patients with any of these features were excluded from the study.

Different laparoscopic techniques have been described for adrenal masses. Each laparoscopic approach has its own advantages and disadvantages. The main difference between individual techniques is between the transperitoneal and retroperitoneal approaches. The choice of the approach depends upon the experience of the surgeon. Several studies have shown no advantage of one approach over the other.[20] The lateral transperitoneal approach allows a wide surgical exposure and inspection of other intra-abdominal organs. In all our patients, we have used this approach because it provides familiar anatomical landmarks, a relatively large working space and allows the viscera to gravitate away from the area of dissection.[21] The retroperitoneal approach carries an elevated risk of hypercapnia and subcutaneous emphysema.[22] However, in case of extensive intra-abdominal adhesions, the retroperitoneal approach may be beneficial.

Robot-assisted adrenalectomy has recently been proposed using the Da Vinci system. Morino *et al*. published a prospective randomised trial with 20 patients, comparing the outcome of Robot-assisted (RA) versus lateral transperitoneal laparoscopic adrenalectomy. This study showed that RA is associated with longer operative time, increased cost and higher morbidity when compared to the lateral transperitoneal approach. However, further studies are needed to define the role of the RA approach.[23]

Mean operating time (150 minutes) is significantly longer for laparoscopic adrenalectomy, but obvious advantages are less morbidity, better cosmesis, reduction in wound infection, minimal need for analgesia, decreased hospital stay and early return to work. In published literature, operating times ranging between 100 and 202 minutes have been reported.[24,25]

The mean hospital stay was four days in our study. Several authors have reported a mean hospital stay of less than three days.[26] The hospital stay could be shortened by admitting the patient on the day of operation and not the day before. We admit all our patients one day before the operation.

Patient no.7 in our series needed conversion to open adrenalectomy because of intra-operative bleeding from the right adrenal vein. Reasons for conversion to open adrenalectomy are usually uncontrollable bleeding, malignancies or widespread adhesions. In other studies, conversion to open surgery has been reported in 3-6%.[22,25]

Intra-operative haemorrhage is the most common complication reported in open and laparoscopic adrenalectomy, accounting for 40% of the overall complications. Other complications include injury to bowel, spleen, pancreas, liver, kidney and diaphragm.[27] The overall complication rate in various reports of laparoscopic adrenalectomy is 9.5%.[28] In our study, none of the patients developed any major complication, except bleeding in one of them, who needed conversion to open. Three patients (13.63%) developed minor complications-port site infection (Patient No.12), chest infection (Patient No.2) and post-operative hypotension (Patient No.14); all were managed conservatively.

In conclusion, laparoscopic adrenalectomy is a safe, effective and useful procedure, without any major post-operative complications and is the current standard of care for all benign adrenal disorders.
